# PBK Enhances Cellular Proliferation With Histone H3 Phosphorylation and Suppresses Migration and Invasion With CDH1 Stabilization in Colorectal Cancer

**DOI:** 10.3389/fphar.2021.772926

**Published:** 2022-01-18

**Authors:** Akira Koshino, Aya Nagano, Akinobu Ota, Toshinori Hyodo, Akane Ueki, Masayuki Komura, Akane Sugimura-Nagata, Masahide Ebi, Naotaka Ogasawara, Kenji Kasai, Yoshitaka Hosokawa, Kunio Kasugai, Satoru Takahashi, Shingo Inaguma

**Affiliations:** ^1^ Division of Gastroenterology, Department of Internal Medicine, Aichi Medical University School of Medicine, Nagakute, Japan; ^2^ Department of Experimental Pathology and Tumor Biology, Nagoya City University Graduate School of Medical Sciences, Nagoya, Japan; ^3^ Department of Biochemistry, Aichi Medical University School of Medicine, Nagakute, Japan; ^4^ Department of Pathology, Aichi Medical University School of Medicine, Nagakute, Japan; ^5^ Department of Pathology, Nagoya City University East Medical Center, Nagoya, Japan

**Keywords:** colorectal cancer, PDZ-binding kinase, phospho-histone H3, E-cadherin, cellular proliferation, migration, invasion

## Abstract

Colorectal cancer (CRC) is one of the most frequent gastrointestinal malignancies with high morbidity and mortality rates. Several biological markers for the prognostication of patient outcome of CRCs are available. Recently, our group identified two favorable factors for the survival of CRC patients: PDZ-binding kinase (PBK) and phospho-histone H3 (PHH3). Both showed a significant inverse association to pT stage. The aim of this study was to uncover the mechanism through which these cellular proliferation–associated protein expressions lead to favorable clinical outcome in CRC patients. We first confirmed co-expression of PBK and PHH3 in CRC cells. Further investigation showed that aberrantly expressed PBK up-regulated the cellular proliferation of CRC cells with accumulation of PHH3. The PBK inhibitor OTS514 suppressed cellular proliferation of CRC cells through down-regulation of PHH3 and induction of apoptosis. *In vitro* studies revealed that PBK suppressed the migration and invasion of CRC cells with suppression of Wnt/β-catenin signaling and CDH1 stabilization. Exogeneous PBK up-regulated the phosphorylated CDH1 at S840, S846, and S847 residues in cultured cells. Recombinant PBK directly phosphorylated HH3; however, it failed to phosphorylate CDH1 directly *in vitro*. The present study demonstrated the association of two markers PBK and PHH3 in CRC. We further identified one of the potential mechanisms by which higher expression of these cellular proliferation–associated proteins leads to the better survival of CRC patients, which likely involves PBK-mediated suppression of the migration and invasion of CRC cells. Our findings suggest that PBK-targeting therapeutics may be useful for the treatment of CRC patients with PBK-expressing tumors.

## Introduction

Multiple biomarkers have been identified to assist in disease diagnosis and predict treatment efficacy and patient outcomes for cancers such as colorectal cancer (CRC) (Oh and Joo, 2020). Cellular proliferation markers such as Ki-67 have been widely used as biomarkers to predict recurrence, treatment response, and prognosis of patients with many types of cancers (Tao et al., 2017; Ahn et al., 2018; Poulakaki et al., 2018; Menon et al., 2019; Xiong et al., 2019). Recently, our group identified two cellular proliferation-associated markers predicting favorable survival for CRC patients; one is PDZ-binding kinase (PBK) (Nagano-Matsuo et al., 2021), and the other is phospho-histone H3 (PHH3) (Koshino et al., 2021).

PBK (also known as TOPK) was initially identified as a kinase that binds to the PDZ domain of DLG1, the human homologue of the *Drosophila* Discs-large (Dlg) (Gaudet et al., 2000). DLG1 is a member of the membrane-associated guanylate kinase homolog (MAGUK) protein family interacting with CDH1 through APC to regulates epithelial cell polarity (Zhang et al., 2011; Roberts et al., 2012). Previous studies revealed that PBK is involved in cytokinesis and spermatogenesis (Gaudet et al., 2000; Matsumoto et al., 2004; Fujibuchi et al., 2005; Abe et al., 2007; Park et al., 2010). PBK has also been reported to enhance the malignant potential of cells, such as by promoting cellular proliferation of tumor cells, and its expression in malignant tumors such as multiple myeloma is associated with patient survival (Brown-Clay et al., 2015; Kwon et al., 2016; Ohashi et al., 2016; Ohashi et al., 2017; Yang et al., 2017; Ota et al., 2020). Several reports have also provided evidence for the anticancer effects of PBK inhibitors on cancer cells (Yang et al., 2016; Herbert et al., 2018; Gao et al., 2019; Zhao et al., 2019). Recently, our group identified a significant inverse correlation between PBK immunohistochemical expression and pT stage in 269 CRCs. Furthermore, we identified high PBK expression in tumor cells as one of the potential favorable factors for CRC patients (Nagano-Matsuo et al., 2021).

PHH3, which is expressed during late G2 and M phases (Pines and Hunter, 1991; Hendzel et al., 1997; McGarry and Kirschner, 1998), has been used for the prognostication of patient outcome, assessment of recurrence risk, and response to treatment in various cancers (Joshi et al., 2015; Tökés et al., 2015). While many types of tumors with high PHH3 expression have been associated with a worse clinical outcome (Ribalta et al., 2004; Ladstein et al., 2012; Nakashima et al., 2013; Nowak et al., 2014; Ramani et al., 2015), our group identified PHH3 as a favorable predictor for the survival of CRC patients (Koshino et al., 2021). However, the mechanism underlying this observation has not been fully elucidated.

In this study, we assessed the association between PBK and PHH3 expressions in CRC cells and examined the mechanism through which these cellular proliferation–associated proteins lead to favorable clinical outcomes in CRC patients.

## Materials and Methods

### Tissue Samples, Clinical Information, and Immunohistochemistry for PBK and PHH3

The Institutional Ethical Review Board of Aichi Medical University Hospital approved this project (2020-H122). The study was performed in compliance with the Helsinki Declaration.

Formalin-fixed paraffin-embedded (FFPE) samples of primary colorectal tumors were obtained as previously described (Inoue et al., 2019; Inoue et al., 2020; Koshino et al., 2021; Nagano-Matsuo et al., 2021). Immunohistochemistry data for PBK, PHH3 and other cellular proliferation markers in 269 CRCs were obtained from our previous reports (Koshino et al., 2021; Nagano-Matsuo et al., 2021). Briefly, FFPE samples of 269 CRCs resected at the Aichi Medical University Hospital from 2009 to 2012 were collected, along with patients’ clinical information (Supplementary Table S1). After surgery, patients were followed for up to 90 months. All CRCs were diagnosed as invasive and naïve to chemotherapy or radiotherapy. A single 4.5-mm core tumor tissue sample derived from FFPE specimens was assembled into multiple blocks containing up to 30 samples. All the cores were taken from invasive areas, and approximately 20% of the cores contained the invasive front.

Immunohistochemistry was performed using a Ventana BenchMark XT Automated ISC/ISH staining instrument (Roche Diagnostics, Basel, Switzerland). Primary antibodies are summarized in Supplementary Table S2. Signals were visualized using 3,3′-diaminobenzidine (DAB). PBK and cytokeratin AE1/AE3 immunoreactive areas were evaluated using ImageJ software (NIH, Bethesda, MD, United States). PBK positivity was determined as follows: the PBK-positive area was divided by cytokeratin AE1/AE3–positive area (%). The number of PHH3-positive cells was counted in a high-power field (×400). Sequential double staining was performed using Leica Bond-Max (Leica Biosystems, Bannockburn, IL, United States), and additional antibody was visualized by HistoGreen.

### Cells, Plasmids, and Reagents

FHC, a human colon epithelial normal cells, were purchased from the American Type Culture Collection (ATCC, Manassas, VA) and cultured according to the recommended procedure by the supplier. HEK293T and CRC cells (Caco-2, COLO205, SW480, CW-2, LoVo, SW48 and HCT116) were obtained as reported previously (Inaguma et al., 2017; Inoue et al., 2020). CRC cells were maintained in Dulbecco's Modified Eagle’s Medium (DMEM) supplemented with 10% fetal bovine serum (FBS).

The lentivirus vectors for PBK and the control LacZ were constructed using the CSII-EF-MCS plasmid, which was kindly provided by Dr. H. Miyoshi (RIKEN BioResource Center, Tsukuba, Japan). The pcDNA3.1 vector (Invitrogen) was used to express PBK, CDH1^cyt^ (CDH1 cytoplasmic domain, R733 to D882) or firefly luciferase (Luc2CP). The CDH1^cyt^ mutants (CDH1^cyt/S840,846,847A^ and CDH1^cyt/S840,846,847D^) were generated by PCR-based mutagenesis. The pGEX-5X-1 vector was used for the synthesis and purification of glutathione S-transferase (GST)-tagged proteins.

OTS514, a selective PBK inhibitor, was purchased from Selleck Biotech (Tokyo, Japan) and used to treat cells for 24–72 h. Colchicine was obtained from FUJIFILM Wako Pure Chemical Corporation (Osaka, Japan) and added at a concentration of 1 × 10^–7^ M for 8 h. MG132, a proteasome inhibitor, was from Selleck Biotech (Tokyo, Japan) and applied at 1 µM for 8 h.

### Cell Cycle Analyses

5 × 10^4^ CRC cells were seeded on 6-well plates. After colchicine treatment at 1 µM for 8 h, cells were harvested, washed and fixed by 70% ethanol for 24 h at −20°C. After saining by the Guava® Cell Cycle Reagent (Guava Technologies, Inc., Hayward, CA), cell cycle analyses were performed using Guava® easyCyte™ systems (Guava Technologies, Inc., Hayward, CA) according to the manufacturer’s protocol.

### Cellular Proliferation Assays

5 × 10^3^ LoVo and CW-2 cells with or without exogeneous PBK expression were seeded on 12-well plates. After incubation, cell numbers were measured using CellTiter 96® Aqueous One Solution (Promega, Madison, WI, United States) according to the manufacturer’s protocol. To assess the suppressive effects of OTS514, 5 × 10^3^ CRC cells on 12-well plates were treated by OTS514 as follows: SW48 for 24 h; HCT116 and LoVo for 48 h.

### Annexin V Assays

To assess the apoptosis induction by OTS514, 5 × 10^4^ CRC cells on 6-well plates were treated by 20 nM of OTS514 as follows: SW48 for 8 h; HCT116 and LoVo for 48 h. Annexin V assays were performed using the Guava Nexin® Reagent (Guava Technologies, Inc., Hayward, CA) and Guava® easyCyte™ systems according to the manufacturer’s protocol.

### Migration and Invasion Assays

Migration and invasion assays were performed using the Falcon® Permeable Support for 24-well Plate with 8.0 µm Transparent PET Membrane and Corning® BioCoat™ Matrigel® Invasion Chambers with 8.0 µm PET Membrane (Corning, NY, United States) according to the manufacturer’s procedure. Cell numbers and incubation times were as follows: migration assays, 2 × 10^4^ cells per chamber and 24 h; invasion assays, 5 × 10^4^ cells per chamber and 48 h. We used 10% FBS as a chemoattractant. After incubation, the migrated or invaded cells from upper chamber to the opposite side of the PET Membrane were fixed using 100% methanol at room temperature and stained with a Giemsa stain. The number of migrated or invaded cells was counted under a microscope.

### Luciferase Reporter Assay

TOP/FOP reporter vectors along with the control hRluc vector (Promega) were transfected using Lipofectamine 3000 reagent (Invitrogen). Dual luciferase reporter assay was performed using the Dual-Luciferase® Reporter Assay System (Promega) according to the manufacturers’ protocol.

### Immunoprecipitation and Immunoblot Analyses

Transfected HEK293T cells were lysed with buffer containing 50 mM Tris, pH 7.5, 5 mM EDTA, 150 mM NaCl, and 0.1% Triton X-100 with protease and phosphatase inhibitor cocktail (Roche, Nutley, NJ, United States). FLAG-tagged proteins were immunoprecipitated using anti-DYKDDDDK tag antibody magnetic beads (FUJIFILM). Whole cell lysates or immunoprecipitated proteins were subjected to immunoblot analyses as previously described (Inaguma et al., 2011; Inaguma et al., 2013). In brief, whole cell lysates were prepared using 1x Sodium Dodecyl Sulfate (SDS) sample buffer, containing 50 mM Tris-HCl and 2% SDS. The SDS polyacrylamide gel electrophoresis was performed using polyacrylamide gel and separated proteins were transferred to a PVDF membrane. Antibody dilutions are summarized in Supplementary Table S2. For sequential detection, antibody stripping buffer (0.1 M Glycine-HCl pH 2.5) was used. Signal intensity was measured by ImageJ software (National Institutes of Health, Bethesda, MD).

### *In Vitro* Kinase Assay

Recombinant PBK and HH3.1 proteins were purchased from Carna Biosciences, Inc. (Kobe, Japan) and New England Biolabs Japan Inc. (Tokyo, Japan), respectively. GST-tagged proteins used in the kinase assays were produced in *Escherichia coli* and purified by the MagneGST™ Protein Purification System (Promega). *In vitro* kinase reactions were performed in 1x kinase buffer (Cell Signaling) with adenosine triphosphate (ATP) labelled by γ-^32^P (PerkinElmer Japan Co., Ltd., Yokohama, Japan) for 90 min at 30°C. After SDS polyacrylamide gel electrophoresis, the signals from phosphorylated proteins were detected by using Fujifilm BAS-5000 (FUJIFILM, Tokyo, Japan).

### Statistical Analysis

All statistical analyses were performed with EZR software version 1.41 (Kanda, 2013). The Mann–Whitney test, the Kruskal–Wallis test with a post-hoc test, or Spearman’s rank correlation coefficient was performed to analyze the significance. *p* < 0.05 indicated statistical significance.

## Results

### PBK Correlated With PHH3 in CRC Cells

**FIGURE 1:**
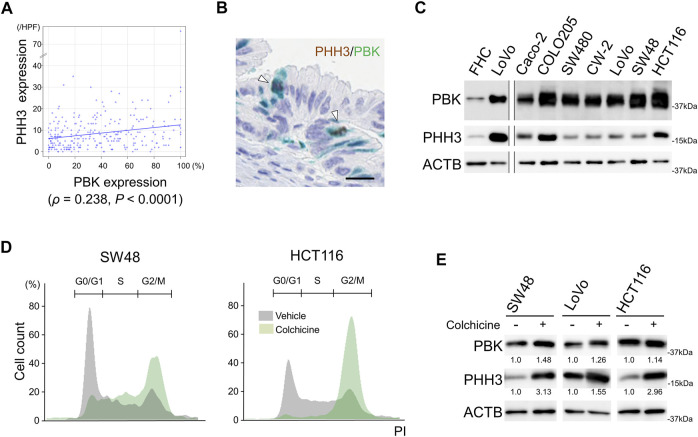
Correlation of PBK with PHH3 in CRC cells. **(A)**, immunohistochemical analysis showing the association of PBK and PHH3 expression in FFPE samples of 269 CRCs. **(B)**, co-expression of PBK and PHH3 in the CRCs during M phase. Bar = 20 μm **(C)**, immunoblot analysis showing the co-expression of PBK and PHH3 in FHC, a normal colonic epithelial cell line, and seven CRC cell lines. **(D)**, cell cycle analysis after colchicine treatment in SW48 and HCT116 cell lines and **(E)**, protein expression of PBK and PHH3 in CRC cells after colchicine treatment. Numbers below the immunoblot bands indicate relative expression.

Immunohistochemical staining analyses revealed that PBK expression showed a significant correlation to PHH3 expression in CRC cells (*ρ* = 0.238, *p* < 0.0001, Figure 1A). PBK expression was also significantly associated with the expression of other cellular proliferation markers (Supplementary Figure S1). The sequential immunohistochemical staining revealed co-expression of PBK and PHH3 in cells in M phase (Figure 1B and Supplementary Figure S2). All of the cultured CRC cells expressed PBK with correlation to PHH3. FHC, a human colonic epithelial cells, expressed PBK and PHH3 at lower levels than CRC cells (Figure 1C). To test the cell cycle-specific expression of PBK and PHH3, we performed colchicine treatment in actively proliferating CRC cells. Colchicine treatment significantly increased M phase around 2.5 folds (*p* < 0.01, Table 1). Cell cycle arrest at M phase by colchicine treatment resulted in up-regulation of both PBK and PHH3 (Figures 1D,E).

**TABLE 1:** Cell cycle analyses in colchicine-treated colorectal cancer cells.

Cell cycle phase	Sub-G0 (%)	G0/G1 (%)	S (%)	G2/M (%)
SW48				
Vehicle	0.1 ± 0.1**	61.9 ± 1.0**	19.1 ± 0.5**	18.9 ± 0.9**
Colchicine	4.8 ± 1.4	18.0 ± 1.3	29.2 ± 0.9	48.0 ± 3.4
LoVo				
Vehicle	1.3 ± 0.2	41.1 ± 1.4**	27.6 ± 1.4**	30.1 ± 1.5**
Colchicine	1.7 ± 0.2	11.4 ± 0.6	14.0 ± 0.1	72.8 ± 0.8
HCT116				
Vehicle	0.4 ± 0.1**	42.3 ± 0.7**	24.3 ± 0.6**	33.5 ± 0.4**
Colchicine	1.1 ± 0.2	4.2 ± 0.3	6.9 ± 0.6	87.9 ± 0.9

**p < 0.01.

### PBK Enhanced Cellular Proliferation With PHH3 Up-Regulation in CRC Cells

**FIGURE 2:**
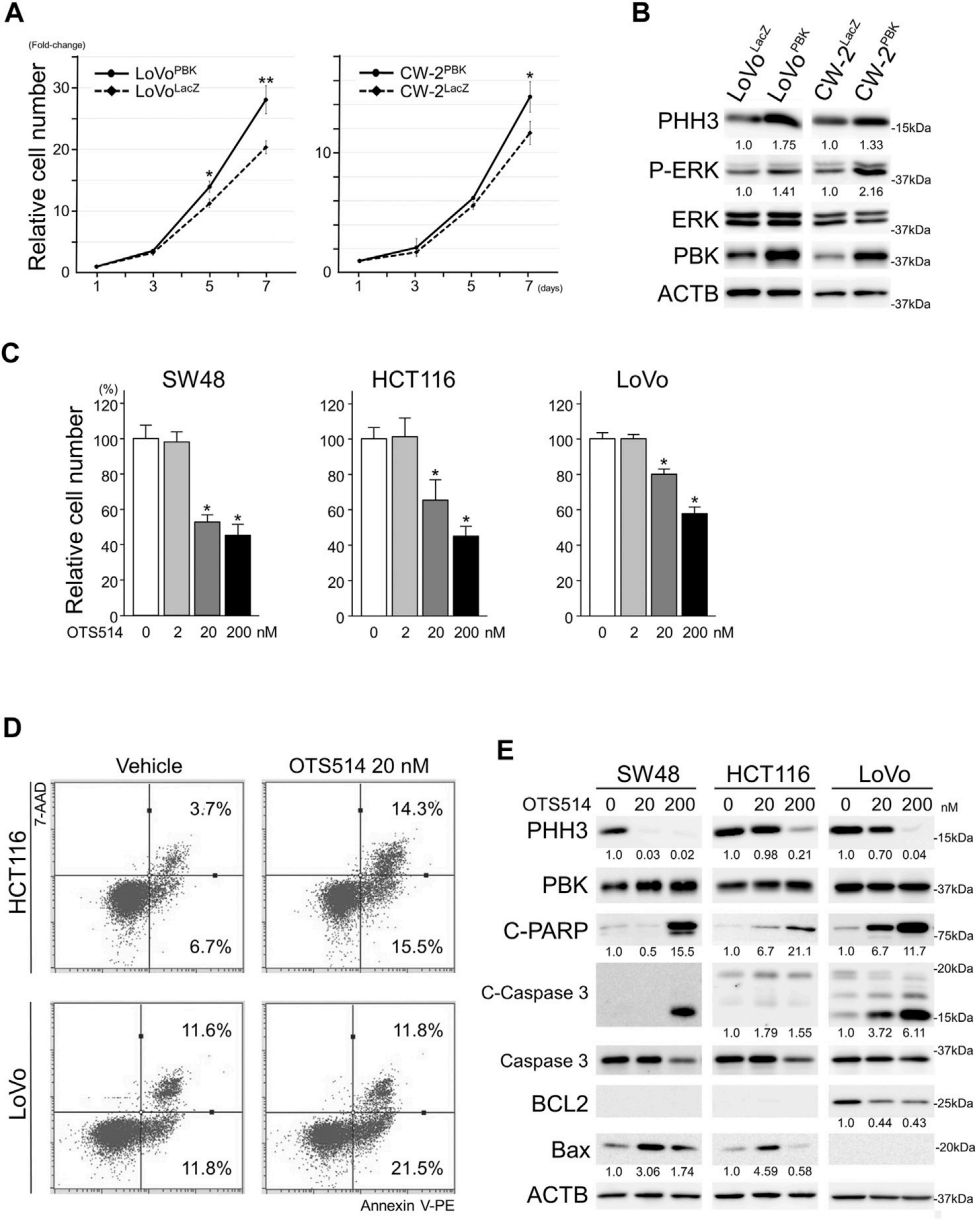
PBK enhanced the cellular proliferation of CRC cells. **(A)**, exogenous PBK expression enhanced the cellular proliferation of CRC cells. Data are shown as mean ± S.D. *, *p* < 0.05, **, *p* < 0.01. **(B)**, immunoblot analysis showing up-regulated P-ERK and PHH3 in PBK-induced CRC cells. **(C)**, OTS514, a selective PBK inhibitor, suppressed the cellular proliferation of CRC cells. Data are shown as mean ± S.D. *, *p* < 0.05. **(D)**, Annexin V assay in HCT116 and LoVo cell lines showing induction of apoptosis after OTS514 treatment. **(E)**, protein expression of PHH3 and apoptosis-related proteins in CRC cells after OTS514 treatment. Note that BCL2 was expressed at under detectable levels in SW48 and HCT116. Bax expression was under detectable level in LoVo. Numbers below the immunoblot bands indicate relative expression.

To uncover the effects of PBK on cellular proliferation, we established CRC cells over-expressing PBK. Forced expression of PBK in LoVo and CW-2 cells significantly enhanced cell proliferation, and these cells also showed PHH3 up-regulation and phospho-ERK accumulation (Figures 2A,B). In contrast, OTS514, a selective PBK inhibitor, significantly suppressed cellular proliferation of CRC cells (Figure 2C). Annexin V assay revealed apoptotic induction in OTS514-treated CRC cells: 1.6 to 2.3 fold increase in early apoptosis (*p* < 0.01) in SW48, HCT116 and LoVo; 1.9 and 4.2 fold increase in late apoptosis (*p* < 0.01) in SW48 and HCT116, respectively (Table 2 and Figure 2D). PHH3 was down-regulated by OTS514 treatment. In contrast, cleaved PARP and cleaved caspase 3 were up-regulated with BCL2 down-regulation or BAX up-regulation by OTS514 (Figure 2E).

**TABLE 2:** Annexin-V Assay in OTS514-treated colorectal cancer cells.

OTS514 (nM)	Early apoptosis (%)		Late apoptosis (%)	
	0	20	0	20
SW48	17.6 ± 0.6	29.5 ± 0.4**	4.6 ± 0.2	8.8 ± 0.9**
HCT116	6.5 ± 0.2	15.1 ± 1.4**	3.5 ± 0.2	14.8 ± 2.4**
LoVo	11.1 ± 0.8	24.7 ± 3.1**	12.5 ± 0.8	14.9 ± 2.9

**p < 0.01.

### PBK Reduced Migration and Invasion of CRC Cells With Stabilizing CDH1

**FIGURE 3:**
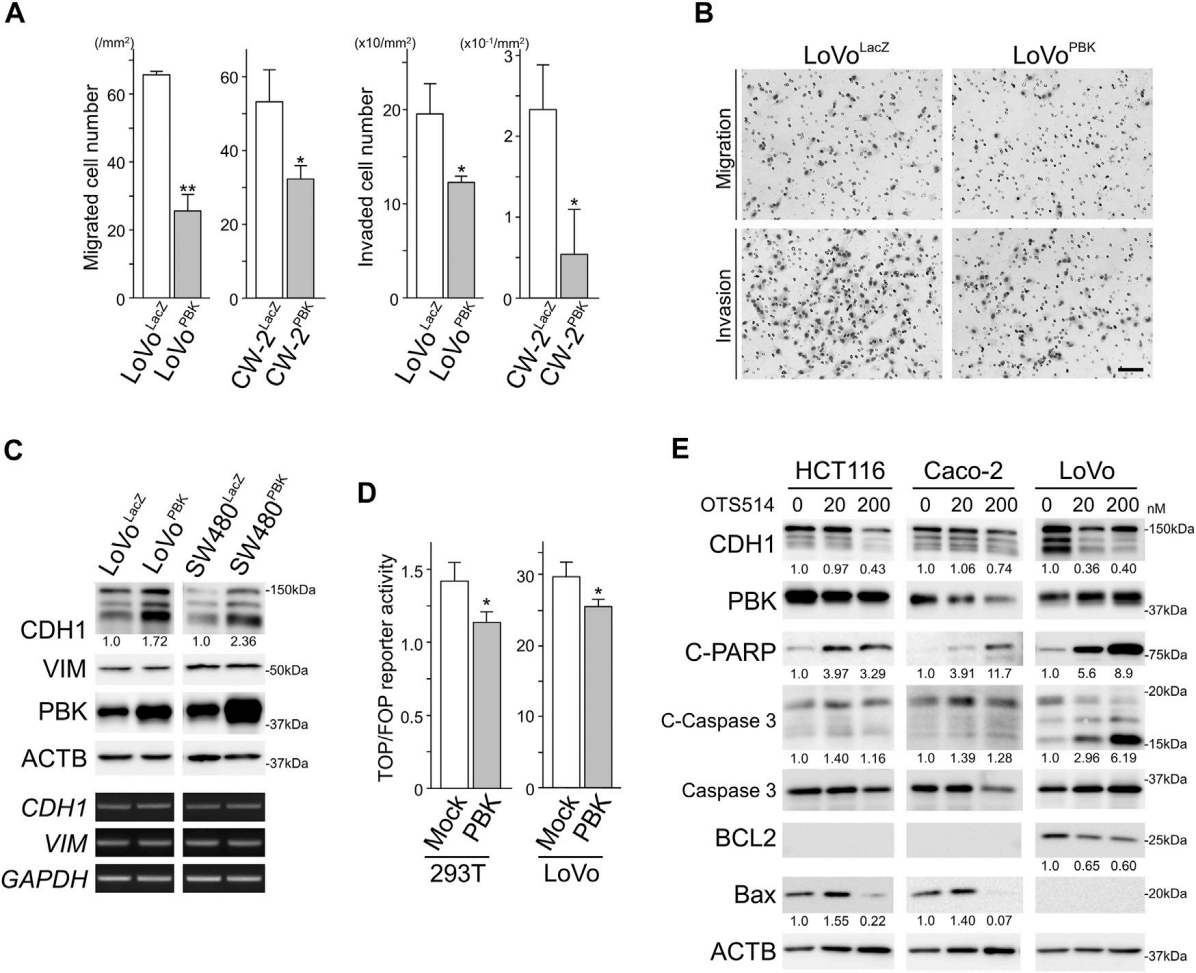
PBK suppressed the migration and invasion of CRC cells with stabilizing CDH1. **A and B**, results of migration and invasion assays showing exogeneous expression of PBK suppressed the migration and invasion of CRC cells. Bar = 100 µm. Assays were performed in triplicate. Data are shown as mean ± S.D. *, *p* < 0.05, **, *p* < 0.01. **(C)**, immunoblot and RT-PCR analysis showing up-regulated CDH1 without altering *CDH1* in PBK induced CRC cells. **(D)**, PBK suppressed the TOP/FOP reporter activity in CRC cells. Assays were performed in triplicate. Data are shown as mean ± S.D. *, *p* < 0.01. **(E)**, protein expression of CDH1 and apoptosis-related proteins in CRC cells after OTS514 treatment. Note that BCL2 was expressed at under detectable levels in HCT116 and Caco-2. Bax expression was under detectable level in LoVo. Numbers below the immunoblot bands indicate relative expression.

Transwell migration and invasion assays revealed that PBK significantly suppressed the migration and invasion activity of CRC cells (Figures 3A,B). To uncover the mechanism by which PBK suppressed the migration and invasion of CRC cells, we analyzed the expression of CDH1 and VIM, those are markers for epithelial-mesenchymal transition (EMT). As shown in Figure 3C, PBK up-regulated CDH1 expression without altering *CDH1* levels, while no changes in VIM or *VIM* was observed. These results indicated that PBK suppressed the migration and invasion of CRC cells through other than EMT. In agreement with CDH1 stabilization, TOP/FOP reporter activities, reflecting internal Wnt/β-catenin signaling activity, was significantly reduced in PBK-transfected cells (Figure 3D). The selective PBK inhibitor OTS514 suppressed CDH1 expression with inducing apoptosis (Figure 3E).

### PBK Phosphorylates the CDH1 Cytoplasmic Domain and HH3

**FIGURE 4:**
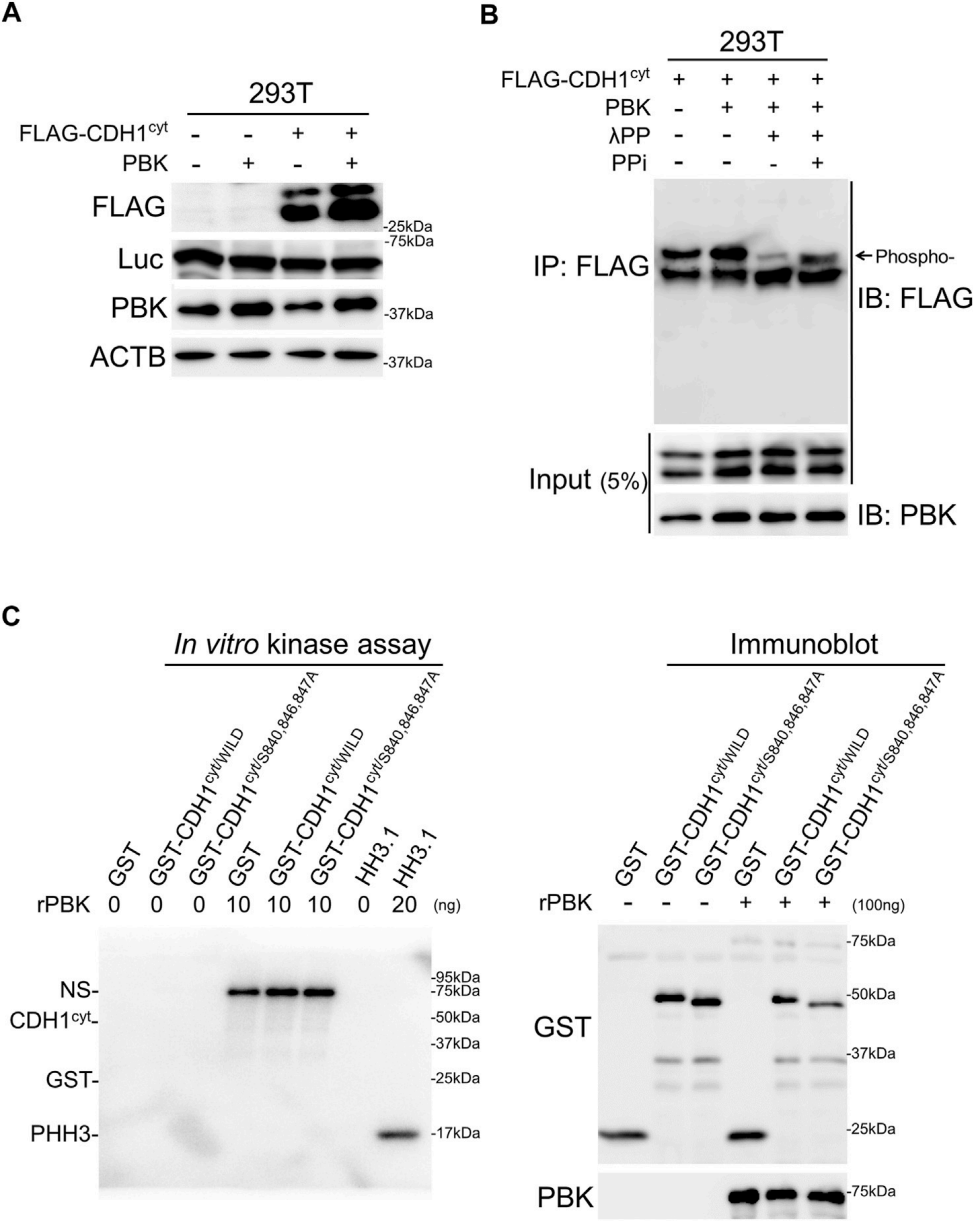
PBK accumulated CDH1 of phosphorylated form and phosphorylated histone H3. **A and B**, immunoblot analysis showing PBK accumulated CDH1^cyt^ with phosphorylation. Note that the expression levels of co-transfected Luc were not affected by PBK. **(C)**, *in vitro* kinase assay showing that both GST-tagged CDH1^cyt/WILD^ and CDH1^cyt/S840,846,847A^ were not phosphorylated by recombinant PBK (rPBK). In contrast, rHH3.1 was directly phosphorylated by rPBK (left panel). Immunoblot analysis showing CDH1^cyt/WILD^ and CDH1^cyt/S840,846,847A^ with or without kinase reaction. Note that no band shift was detected after kinase reaction (right panel). NS, non-specific.

PBK was initially identified as a kinase that binds to the PDZ domain of DLG1, a member of the MAGUK protein family (Gaudet et al., 2000), which was reported to interact with CDH1 through APC and regulates epithelial cell polarity (Zhang et al., 2011; Roberts et al., 2012). Therefore, we assessed the possibility that PBK stabilizes CDH1 via phosphorylation of the CDH1 cytoplasmic domain (CDH1^cyt^) (McEwen et al., 2014). To test this possibility, an expression vector for CDH1^cyt^ was co-transfected with vectors encoding Luc2CP and PBK. PBK stabilized CDH1^cyt^ with accumulation of its phosphorylated form (Figures 4A,B, and Supplementary Figure S3A). The expression level of co-transfected Luc2CP was not affected by PBK, supporting the notion that PBK stabilized CDH1^cyt^ protein not but activated CMV promoter of the expression vectors. In addition, CDH1^cyt^ accumulation was not from the down-regulation of proteasome activity (Supplementary Figure S3A).

To assess the direct CDH1^cyt^ phosphorylation by PBK, *in vitro* kinase assays were performed using GST-tagged proteins synthetized in *E. coli* (Supplementary Figure S3B). As shown in Figure 4C left panel and Supplementary Figure S3C, both GST-tagged CDH1^cyt/WILD^ and CDH1^cyt/S840,846,847A^ were not phosphorylated by recombinant PBK. Furthermore, no band shifts were detected in both CDH1^cyt/WILD^ and CDH1^cyt/S840,846,847A^ after the kinase reaction (Figure 4C, right panel). These results indicated that PBK accumulates CDH1 with S840, S846, and S847 phosphorylation; however, PBK itself may not phosphorylate CDH1 directly (McEwen et al., 2014). In contrast to CDH1, *in vitro* kinase assay revealed that recombinant PBK directly phosphorylated recombinant HH3.1 (Figure 4C, left panel and Supplementary Figure S3C) consistent with the previous report (Park et al., 2006).

## Discussion

In the present study, we showed the significant association between PBK and PHH3 expressions in CRC, both of which are favorable factors for the survival of CRC patients (Koshino et al., 2021; Nagano-Matsuo et al., 2021). We further identified one of the potential mechanisms by which higher expression of these cellular proliferation–associated proteins leads to the better survival of CRC patients: PBK-mediated suppression of the migration and invasion of CRC cells via CDH1 stabilization.

In contrast to reports in malignant tumors such as multiple myeloma in which PBK enhances the malignant potential of tumor cells and is associated with unfavorable clinical outcome (Brown-Clay et al., 2015; Kwon et al., 2016; Ohashi et al., 2016; Ohashi et al., 2017; Yang et al., 2017; Ota et al., 2020), our study revealed favorable survival of CRC patients with PBK-high tumors (Nagano-Matsuo et al., 2021). PBK showed a significant positive association with Ki-67, a widely used cellular proliferation marker, along with a significant inverse association to pT stage in CRC (Nagano-Matsuo et al., 2021). We thus hypothesized that PBK up-regulates cellular proliferation while it suppresses migration or invasion in CRC cells. We performed *in vitro* experiments using CRC cell lines and our results support this hypothesis. Whether PBK-dependent CDH1 stabilization and suppression of migration and invasion activity is a CRC cell–specific phenomena is not clear; however, other epithelial tumors in which *PBK*-high predicts favorable clinical outcome might show the same phenomena as CRC (Supplementary Table S3). Further investigation may uncover additional organ- or tumor type–specific PBK functions.

CDH1 (E-cadherin) is the core component of epithelial adherens junctions, which are essential for tissue development, differentiation, and maintenance. CDH1 serves as a critical tumor suppressor that tethers β-catenin, one of the key molecules for Wnt/β-catenin signaling, around the cytomembrane to suppress its nuclear translocation and transcriptional activation. CDH1 insufficiency has been linked to malignant activities such as invasiveness, cellular proliferation, and anti-apoptosis (Wong et al., 2018). CDH1 functions are regulated in part by the phosphorylation of specific amino acid residues located within its cytoplasmic domain. Phosphorylation of T790 in CDH1 by protein kinase Cδ reduces CDH1 function, allowing β-catenin nuclear translocation and transcription (Chen et al., 2016), while phosphorylation of S840, S846, and S847 in CDH1 enhances β-catenin tethering, cell adhesion, and stability of surface CDH1 (McEwen et al., 2014). However, the kinase(s) responsible for the phosphorylation of S840, S846, and S847 residues have not been fully uncovered. Notably, our results identified PBK as a potential kinase that phosphorylates the S840, S846, and S847 of CDH1 and stabilizes CDH1 protein; however, PBK was not supposed to phosphorylate CDH1 directly (Figure 4). Kinase(s) responsible for the phosphorylation of the S840, S846, and S847 of CDH1 should be elucidated in the near future.

Histones are highly basic proteins that are found in eukaryotic cell nuclei, and an octamer of core histones (H2A, H2B, H3 and H4) composes nucleosomes. Nucleosomes not only serve as spools for genomic DNA but also are crucial for biological processes including gene replication and gene transcription (Tessarz and Kouzarides, 2014). Biochemical modifications, such as methylation, acetylation, and phosphorylation, of specific amino acid residues of histones have been reported to be critical in these physiological processes, and disruption of these modifications has been reported to have roles during malignant transformation (Komar and Juszczynski, 2020). PHH3, expressed during late G2 and M, has been used as a specific immunohistochemical indicator of proliferating cells in FFPE sections. In diagnostic pathology, PHH3 is considered as a potential immunohistochemical marker for grading, prognostication, and assessment of recurrence risk and as an indicator of the response to treatment of patients with malignancies (Ribalta et al., 2004; Ladstein et al., 2012; Nakashima et al., 2013; Nowak et al., 2014; Ramani et al., 2015). In contrast to other malignancies in which higher expression of PHH3 predicts worse clinical outcome (Ribalta et al., 2004; Ladstein et al., 2012; Nakashima et al., 2013; Nowak et al., 2014; Ramani et al., 2015), PHH-high CRCs showed favorable outcome (Koshino et al., 2021). Consistent with previous report (Park et al., 2006), our results showed that PBK directly phosphorylated HH3. Furthermore, PBK suppressed the migration and invasion of CRC cells. These observations may partly explain our previous discrepant results (Koshino et al., 2021), in which favorable clinical outcome and inverse association to pT stage in PHH3-high CRC, is from upstream PBK functions. Previous studies reported that gene mutations or variants of histones lead to carcinogenesis (Komar and Juszczynski, 2020). Whether PBK-phosphorylated HH3 is a marker for cellular proliferation or it harbors malignant potential during CRC carcinogenesis is unclear.

PBK has been considered as a promising therapeutic target and multiple studies have been conducted to target this gene (Herbert et al., 2018). Several PBK-targeting compounds such as HI-TOPK-032, SKLB-C05 and Ginsenoside Rh2 have been reported to suppress tumor growth and/or metastasis of CRC (Gao et al., 2019; Yang et al., 2016; Kim et al., 2012). Our group successfully inhibited the growth of multiple myeloma and CRC cells using OTS514, a specific PBK inhibitor (Figures 2C,D) (Ota et al., 2020; Nagano-Matsuo et al., 2021). OTS514 not only suppressed the cellular proliferation of CRC cells but it also induced apoptosis with down-regulation of PHH3. Based on the PBK-dependent cellular proliferation (Figure 2), PBK-targeting therapeutics may be an effective strategy for the treatment of CRC patients. One concern is whether PBK inhibition enhances the migration and invasion of CRC cells; however, we consider that PBK inhibition will lead to apoptosis rather than enhancement of migration and invasion (Figure 3E). In addition, toxic side effects on PBK-expressing organs such as testis should be carefully assessed before clinical application.

The present study demonstrated an association of PBK and PHH3 expressions in CRC, both of which are immunohistochemical markers for favorable clinical outcomes in CRC patients. PBK-mediated up-regulation of cellular proliferation and suppression of migration and invasion of CRC cells were also demonstrated. These observations may partly explain discrepancies observed in our past studies (Koshino et al., 2021; Nagano-Matsuo et al., 2021) in which CRCs with higher proliferative activity resulted in favorable clinical outcome. Furthermore, PBK-targeting therapies may be candidate strategies for treating CRC patients.

## Publisher’s Note

All claims expressed in this article are solely those of the authors and do not necessarily represent those of their affiliated organizations, or those of the publisher, the editors and the reviewers. Any product that may be evaluated in this article, or claim that may be made by its manufacturer, is not guaranteed or endorsed by the publisher.

## Data Availability

The raw data supporting the conclusions of this article will be made available by the authors, without undue reservation.
