# Supplementary material for: PBK Enhances Cellular Proliferation With Histone H3 Phosphorylation and Suppresses Migration and Invasion With CDH1 Stabilization in Colorectal Cancer
Source: Front Pharmacol. 2022 Jan 18;12:772926. doi: 10.3389/fphar.2021.772926 (PMC8804381; doi:10.3389/fphar.2021.772926)
Supplement: Supplementary file 1 [file DataSheet1.docx]

Supplementary Material

**SUPPLEMENTARY METHODS**

**Association of PBK expression and cellular proliferation marker labelling indices**

Labelling indices for CCNA, GMNN, and Ki-67 were determined by counting > 500 tumor cells per case in a high-power field (HPF, ×400) in our previous study [[8](#_ENREF_8)]. Spearman’s rank correlation coefficient was performed to analyze the significance.

**Survival analyses in Kaplan–Meier Plotter**

Survival analyses were performed using the Kaplan–Meier Plotter program Pan-cancer RNA-sec data according to *PBK* expression (<https://kmplot.com/analysis/>). The best cut-off values were automatically set by the program in each tumor type.

**SUPPLEMENTARY TABLES**

| **Supplementary Table S1. Characteristics of Colorectal Cancer Patients** | | | | |
| --- | --- | --- | --- | --- |
|  |  |  |  |  |
|  |  | Total No. | |  |
|  |  | 269 | (100%) |  |
| Sex | |  |  |  |
|  | Male | 143 | [53%] |  |
|  | Female | 126 | [47%] |  |
|  |  |  |  |  |
| Age, years (mean±S.D.) | | 68.6±12.6 | |  |
|  | |  | |  |
| Size, cm (mean±S.D.) | | 5.0±2.6 | |  |
|  | |  |  |  |
| Tumour location | |  |  |  |
|  | Right-sided colon | 124 | [46%] |  |
|  | Left-sided colon | 86 | [32%] |  |
|  | Rectum | 59 | [22%] |  |
|  | |  |  |  |
| pT stage | |  |  |  |
|  | pT2 | 36 | [13%] |  |
|  | pT3 | 189 | [70%] |  |
|  | pT4 | 44 | [16%] |  |
|  | |  |  |  |
| Histological differentiation | |  |  |  |
|  | Well to moderately | 242 | [90%] |  |
|  | Poorly | 27 | [10%] |  |
|  |  |  |  |  |
| Mucus production | |  |  |  |
|  | Positive | 14 | [5%] |  |
|  | Negative | 255 | [95%] |  |
|  | |  |  |  |
| Lymph node metastasis | |  |  |  |
|  | Positive | 124 | [49%] |  |
|  | Negative | 129 | [51%] |  |
|  | |  |  |  |
| Peritoneal metastasis | |  |  |  |
|  | Positive | 50 | [19%] |  |
|  | Negative | 219 | [81%] |  |
|  |  |  |  |  |
| Distant organ metastasis | |  |  |  |
|  | Positive | 44 | [16%] |  |
|  | Negative | 225 | [84%] |  |
|  |  |  |  |  |
| Operation status | |  |  |  |
|  | Complete resection | 237 | [88%] |  |
|  | Incomplete resection | 32 | [12%] |  |
|  |  |  |  |  |
| MMR system status | |  |  |  |
|  | Deficient | 31 | [12%] |  |
|  | Preserved | 238 | [88%] |  |

Characteristics of the 269 CRC patients are summarized.

|  |  | |  | |  |  | |  |
| --- | --- | --- | --- | --- | --- | --- | --- | --- |
| **Supplementary Table S2. Antibodies and Conditions for Immunohistochemistry and Immunoblotting** | | | | | | | | |
|  | | IHC | | IB | | |  | |
| Genes | | Reagent | Dilution | Dilution | | | Antibodies | |
| ACTB | | - | - | 5,000 | | | AC-74, SIGMA (Rocklin, CA) | |
| Bax | | - | - | 1,000 | | | Clone D2E11, Cell Signaling Technology, Inc. (Danvers, MA) | |
| BCL2 | | - | - | 1,000 | | | Clone D55G8, Cell Signaling Technology, Inc. (Danvers, MA) | |
| Caspase 3 | | - | - | 1,000 | | | #9662, Cell Signaling Technology, Inc. (Danvers, MA) | |
| C-Caspase 3 | | - | - | 1,000 | | | Clone 5A1E, Cell Signaling Technology, Inc. (Danvers, MA) | |
| CCNA | | IV | 100 | - | | | sc-751, Santa Cruz Biothechnology, Inc. (Dallas, TX) | |
| CDH1 | | - | - | 1,000 | | | Clone 24E10, Cell Signaling Technology, Inc. (Danvers, MA) | |
| CK AE1/3 | | IV | 1 | - | | | Clone AE1/3 NICHIREI BIOSCIENCES INC. (Tokyo, Japan) | |
| ERK | | - | - | 1,000 | | | #9102, Cell Signaling Technology, Inc. (Danvers, MA) | |
| P-ERK | | - | - | 1,000 | | | #9101, Cell Signaling Technology, Inc. (Danvers, MA) | |
| FLAG | | - | - | 1,000 | | | M2 HRP-conjugated, Invitrogen/Thermo Fisher Scientific (Tokyo, Japan) | |
| Geminin | | OV | 100 | - | | | EPR14637, Abcam (Cambridge, UK) | |
| GST | | - | - | 1,000 | | | Kindly provided by Dr. Toshinori Hyodo* | |
| Ki-67 | | OV | 100 | - | | | Clone MIB-1, Dako/Agilent (Santa Clara, CA) | |
| Luciferase | | - | - | 10,000 | | | PM016, MBL CO., LTD. (Tokyo, Japan) | |
| MLH1 | | OV | 200 | - | | | Clone G168-728, BD Biosciences (Franklin Lakes, NJ) | |
| MSH2 | | OV | 200 | - | | | Clone G219-1129, BD Biosciences (Franklin Lakes, NJ) | |
| MSH6 | | OV | 400 | - | | | Clone 44/MSH6, BD Biosciences (Franklin Lakes, NJ) | |
| C-PARP | | - | - | 1,000 | | | Clone D64E10, Cell Signaling Technology, Inc. (Danvers, MA) | |
| PMS2 | | OV+Linker | 50 | - | | | Clone A16-4, BD Biosciences (Franklin Lakes, NJ) | |
| PBK | | IV | 100 | - | | | sc-293028, Santa Cruz Biothechnology, INC (Dallas, TX) | |
| PBK | | - | - | 5,000 | | | EPR21983, Abcam (Cambridge, UK) | |
| PHH3 | | OV | 500 | - | | | Cell Marque^TM^, Millipore SIGMA (Rocklin, CA) | |
| PHH3 Ser10 | | - | - | 1,000 | | | ab136810, Abcam (Cambridge, UK) | |
| VIM | | - | - | 1,000 | | | Clone D21H3, Cell Signaling Technology, Inc. (Danvers, MA) | |

IV, iView reagent. OV, OptiView reagent. Antigen retrieval was performed with heat activation in high pH buffer.

*; Hyodo et. al., The Journal of Biological Chemistry, 287（30）, 2012. DOI: 10.1074/jbc.M112.372342

|  |  |  |  | | |  | |  | |  | |
| --- | --- | --- | --- | --- | --- | --- | --- | --- | --- | --- | --- |
| **Supplementary Table S3. Survival analyses in Kaplan-Meier Plotter according to *PBK* expression** | | | | | | | | | | | |
|  |  | | | Patient | Hazard | | 95% CI | | | | Log-rank |
|  |  | | | No. | Ratio | | min | | max | | *P*-value |
| Thymoma | | | | 118 | 0.10 | | 0.02 | | 0.49 | | 0.00065 |
| Oesophageal squamous cell carcinoma | | | | 81 | 0.25 | | 0.11 | | 0.61 | | 0.00088 |
| Cervical squamous cell carcinoma | | | | 304 | 0.52 | | 0.32 | | 0.85 | | 0.0072 |
| Lung squamous cell carcinoma | | | | 495 | 0.66 | | 0.50 | | 0.87 | | 0.0033 |
| Ovarian cancer | | | | 373 | 0.66 | | 0.50 | | 0.87 | | 0.0027 |
| Stomach adenocarcinoma | | | | 371 | 0.75 | | 0.54 | | 1.03 | | 0.078 |
| Lung adenocarcinoma | | | | 504 | 1.74 | | 1.28 | | 2.35 | | 0.00029 |
| Clear cell renal cell carcinoma | | | | 530 | 1.91 | | 1.40 | | 2.60 | | <0.0001 |
| Pancreatic ductal adenocarcinoma | | | | 177 | 2.03 | | 1.30 | | 3.13 | | 0.001 |
| Hepatocellular carcinoma | | | | 370 | 2.05 | | 1.42 | | 2.95 | | <0.0001 |
| Oesophageal adenocarcinoma | | | | 80 | 2.40 | | 1.26 | | 4.58 | | 0.0061 |
| Papillary renal cell carcinoma | | | | 287 | 4.62 | | 2.53 | | 8.44 | | <0.0001 |

RNA-seq data were analyzed using the Kaplan–Meier Plotter program on the web site.

**Supplementary Figure S1. Associations between cellular proliferation marker expressions and PBK expression**.

**
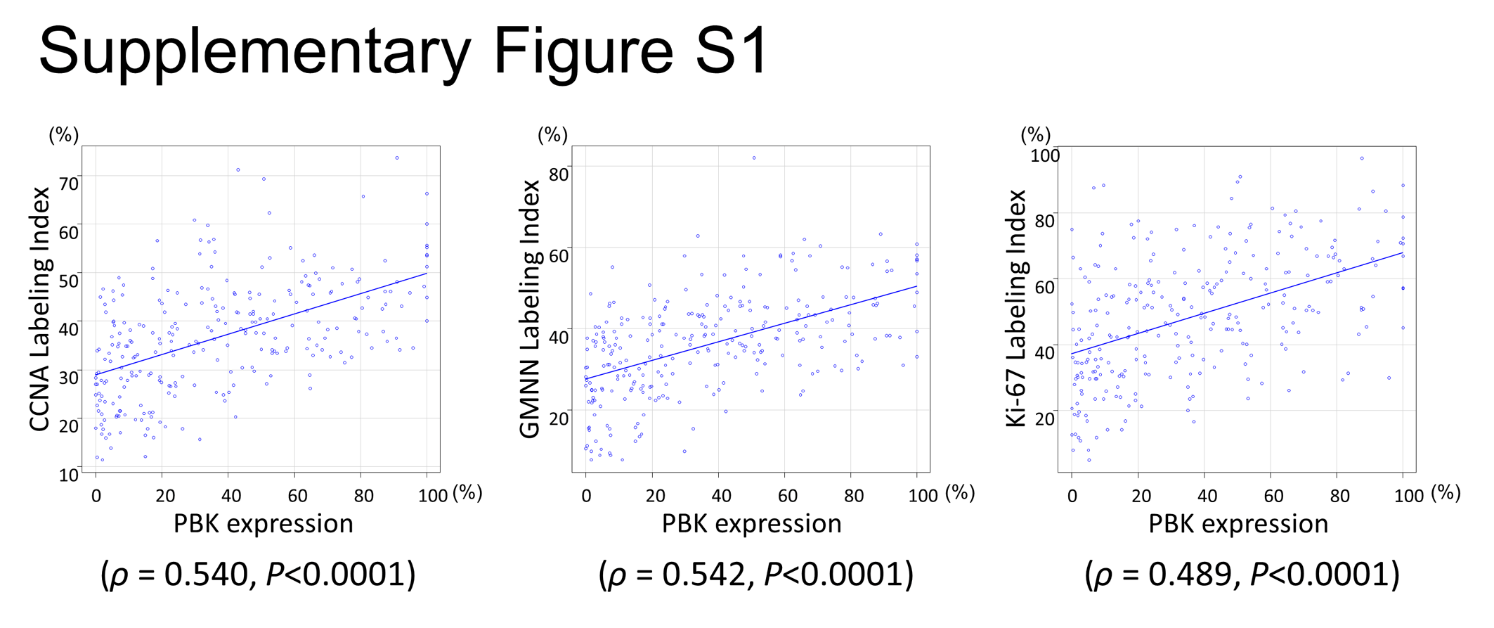
**

CCNA, GMNN, and Ki-67 labelling indices showed significant association to PBK expression.

**Supplementary Figure S2. Co-expression of PBK and PHH3 in CRC cells in M phase**.

**
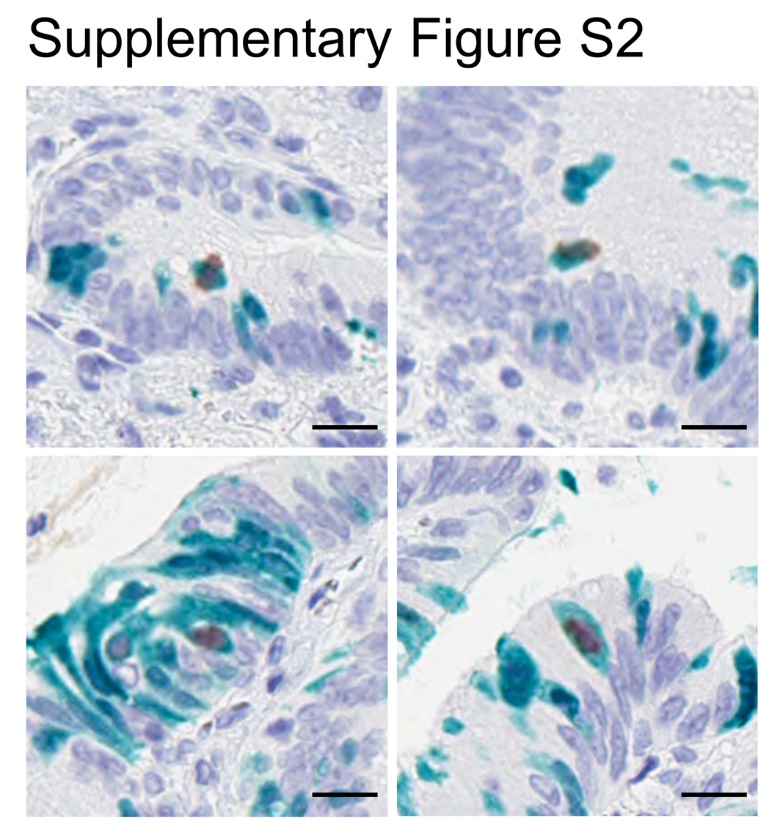
**

In sequential immunostaining, co-expression of PBK and PHH3 was observed. Bar = 20 µm.

**Supplementary Figure S3. SDS-PAGE and immunoblot analyses of FLAG- or GST-tagged CDH1^cyt^ with or without mutations**.

**
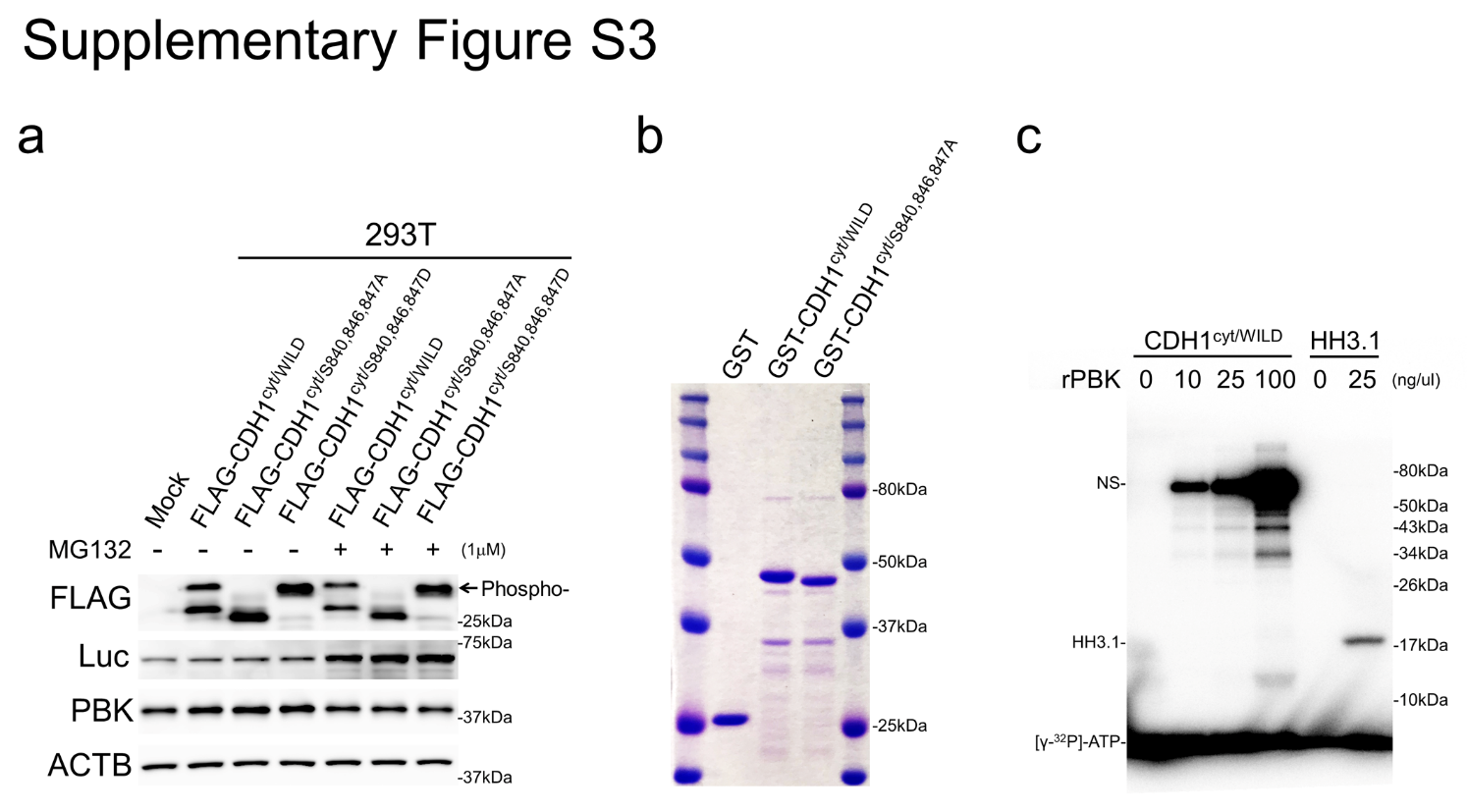
**

a, immunoblot analyses showing specific band shifts of FLAG-CDH1^cyt/S840,846,847A^ and FLAG-CDH1^cyt/S840,846,847D^ mutants. MG132, a proteosome inhibitor, did not affect CDH1^cyt^ expressions. In contrast, Luc2CP was accumulated in the MG132-treated group. b, coomassie Brilliant Blue staining of GST-tagged proteins synthetized in *E. coli*. 500ng of each protein was separated by SDS-page. c, *in vitro* kinase assay revealed that rPBK direct phosphorylated rHH3.1, while it failed to phosphorylate CDH1^cyt/WILD^. NS, non-specific.
